# Influence of Nutrition, Lifestyle Habits, and Socio-Demographic Determinants on Eating Disorder Symptoms in the Spanish Young Adult Population: A Cross-Sectional Nationwide Survey

**DOI:** 10.3390/medicina60101565

**Published:** 2024-09-24

**Authors:** Elena Sandri, Marco Sguanci, Eva Cantín Larumbe, Germán Cerdá Olmedo, Michela Piredda, Stefano Mancin

**Affiliations:** 1 Faculty of Medicine and Health Sciences, Catholic University of Valencia San Vicente Mártir, c/Quevedo, 2, 46001 Valencia, Spain; elena.sandri@ucv.es (E.S.); german.cerda@ucv.es (G.C.O.); 2 Doctoral School, Catholic University of Valencia San Vicente Mártir, c/Quevedo 2, 46001 Valencia, Spain; 3 Department of Medicine and Surgery, Research Unit Nursing Science, Campus Bio-Medico di Roma University, Via Alvaro del Portillo 21, 00128 Rome, Italy; sguancim@gmail.com; 4 Faculty of Data Science, Polytechnical University of Valencia, Camí de Vera s/n, 46022 Valencia, Spain; evacantinlarumbe@gmail.com; 5 Department of Biomedicine and Prevention, University of Rome “Tor Vergata”, Viale Montpellier, 1, 00128 Rome, Italy; stefano.mancin@humanitas.it

**Keywords:** feeding and eating disorders, feeding behavior, healthy lifestyle, survey, Spain

## Abstract

Eating disorders represent a complex and multifaceted public health challenge, highly prevalent among young people. *Background and Objectives*: To examine the prevalence of eating disorders in the Spanish young adult population aged 18–30 years, and their correlation with various eating habits and lifestyle factors. *Materials and Methods*: A descriptive, cross-sectional survey was carried out using a non-probabilistic snowball sample. The valid and reliable NutSo-HH Scale was employed to collect data on nutrition, lifestyle, and health habits. *Results*: Data were collected from 9692 Spanish young adults, of which 101 (1.04%) were diagnosed with anorexia nervosa and 71 (0.73%) with bulimia nervosa. The prevalence of anorexia and bulimia was significantly higher (*p* < 0.001) in women than in men, regardless of socio-demographic variables such as educational level, income, region, and size of city of residence. A considerable percentage of participants showed possible symptoms of eating disorders. Nutritionally, individuals with eating disorders consumed less fast food and fried or ultra-processed food but tended to consume coffee and energy drinks more frequently. Additionally, sleep quality and duration were more adversely affected in individuals with eating disorders compared to the general population. Sedentary lifestyles did not significantly differ between those with eating disorders and healthy individuals, though physical activity increased in people with bulimia. *Conclusions*: The interplay between young adults’ dietary habits, lifestyle factors, and mental health underscores the urgent need for targeted interventions to effectively address these complex public health challenges.

## 1. Introduction

Eating disorders (EDs) including anorexia nervosa (AN), bulimia nervosa (BN), and binge eating disorder represent a major public health challenge with severe implications for both physical and mental well-being [1,2]. These disorders have seen a marked increase since the late 20th century, with the rise in AN, BN, and binge eating disorder posing significant health risks [3]. Historically, the ideal body shape for young women in Western societies was fuller, but since the 1960s, the standard has shifted toward a much slimmer figure. For men, muscular and robust physiques have remained the ideal [1]. These evolving standards, reinforced by media and social networks, often lead to body dissatisfaction and promote extreme dieting and unhealthy weight control practices, contributing to the development of EDs [4]. The rise in ED prevalence over recent decades may also be partly due to improved diagnosis and treatment methods [5].

A systematic review revealed that the prevalence of EDs increased from an average of 3.5% between 2000 and 2006 to 7.8% between 2013 and 2018 [6]. In the U.S., a study found that the highest mean annual prevalence of EDs occurs at around 21 years of age, with 10.3% of females and 7.4% of males affected. Lifetime prevalence estimates rise to 19.7% for females and 14.3% for males by age 40 [7]. EDs are notably more common among younger individuals [8,9], making early intervention crucial. Understanding the patterns of EDs and their links to specific lifestyle and nutritional habits is essential for developing effective prevention and intervention strategies.

Understanding the factors influencing eating disorders is crucial, particularly in young adults, who face unique social, psychological, and lifestyle challenges during the transition from adolescence to adulthood [10,11]. Defining young adulthood varies globally; the United Nations sets it between 15 and 24 years [12], while the World Health Organization defines it as 10–24 years [13]. In Spain, the upper age limit is often extended to 29 or 30 due to societal changes [14,15,16]. Some studies even classify young adults up to age 40 [17,18,19]. The prevalence of eating disorders in Europe is similar to other developed nations [20], and research specific to Spain has highlighted the need for age-focused studies [21,22]. For example, Alfonso-Fuertes et al. [23] found that young adults are particularly vulnerable to body image issues and societal pressures, which may contribute to the onset of eating disorders.

There is extensive research on the influence of sociocultural factors on eating behaviors in Spain [24,25,26]. However, these studies often do not analyze how these factors relate to the prevalence of eating disorders. While they provide valuable insights into the interplay between social expectations and individual perceptions of body image [27,28], they have primarily focused on adolescents or university settings, leaving a gap in research concerning older young adults. For example, Adelantado-Renau [22] examined the association between the risk of EDs and academic performance, and Gómez-Romero [29] explored the link between the risk of developing an eating disorder and suicidal thoughts among adolescents in schools. Other studies have focused on university students, such as Escolar-Llamazares et al. [30], who analyzed the prevalence of the risk of eating disorders and their association with obesity and physical fitness, and Parra-Fernández et al. [31], who studied possible associations between orthorexia and psychological traits in Spanish university students. These studies investigated factors influencing the risk of eating disorders such as gender, age, course of study, degree, and body mass index (BMI) in university students.

A detailed understanding of the influence of nutrition, lifestyle habits, and socio-demographic determinants on the prevalence of eating disorders (EDs) in the young Spanish population is useful for several reasons. These include earlier detection and more effective treatments for affected young people and the identification of specific risk factors, which facilitates the creation of preventive strategies. Studying the impact of factors such as socio-economic status, education, and social environment allows for the design of more effective and personalized intervention programs that are tailored to the socio-demographic and cultural characteristics of young people in Spain. It may also allow the identification of subgroups within the youth population that may be more predisposed to develop EDs.

Finally, in economic terms, the benefits may also be notable, as by identifying and addressing the factors that contribute to ED in youth, more serious long-term health problems may be prevented, reducing health care costs.

### Study Aim

This study aimed to examine the prevalence of eating disorders in the Spanish young adult population aged 18–30 years as well as their correlation with various eating habits and lifestyle factors.

## 2. Materials and Methods

### 2.1. Study Design and Sampling

A cross-sectional study was undertaken to explore the young Spanish population aged 18–30 years residing in Spain. Individuals with chronic diseases or temporary conditions that could impact their diet, such as confinement in a prison or hospital admission, were excluded from the study. Participants were approached via social media or email and encouraged to further share the link to the online survey through the “snowball” method [31]. This study adhered to the STROBE Guidelines [32] (see Supplementary Materials).

### 2.2. Ethics Approval of Research

The research adhered to the principles outlined in the Declaration of Helsinki and received approval from the Research Ethics Committee of the Catholic University of Valencia (approval code UCV/2019-2020/152, dated 18 June 2020). Informed consent was obtained from all participants involved in the study.

### 2.3. Instrument

This study employed the Nutrition and Social Healthy Habits Scale (NutSo-HH) [33], a self-developed and validated questionnaire comprising three sections. The questionnaire investigated: (1) nutrition habits (consumption frequencies of various food groups); (2) health-related social habits (exercise, smoking, alcohol consumption, sleep patterns); and (3) anthropometric (weight, height) and socio-demographic data (sex, age, place of birth and residence, occupation, education, income level). Self-perceived health status and symptoms of eating disorders were also assessed.

The instrument was developed and validated following rigorous methodological approaches. Content validity was assessed by an expert group (N = 7) consisting of a nutritionist, two family doctors, two psychologists, a social educator, and a communication expert. Face validity was evaluated in a pilot study involving 53 individuals with characteristics similar to the study population [33].

### 2.4. Data Collection

The questionnaire was administered using Google Forms, and its distribution primarily leveraged Instagram as the main channel, employing a non-probabilistic snowball sampling approach [31]. This method began with initial participants who met the study’s criteria and then involved them in identifying others who might qualify and be interested, continuing this process until an adequate number of participants was reached. The Instagram account “@elretonutricional” was established for dissemination, engaging various professionals, influencers, and supporters. Researchers also utilized their networks on LinkedIn, Twitter, WhatsApp, and Facebook. Additionally, emails were sent to various establishments across Spain, selected for their diverse clientele including pharmacies and tobacconists. Data collection took place from August 2020 to November 2021.

### 2.5. Variables

Most variables in the study were qualitative, offering respondents multiple options to choose from. These included the frequency of food and drink consumption, sedentary lifestyle, hours of sleep, tobacco use, sex (male or female), place of birth and residence, and level of education. Education was categorized as basic (including no education, primary or secondary education, vocational training, or baccalaureate) and higher (including bachelor’s, master’s, or doctoral degrees). Income level was measured per household (EUR <2200 per month: low income; EUR >2200 per month: medium-high income).

Quantitative continuous variables included age, weight, height (self-reported), and the number of minutes engaged in sports per week. Other variables were discretely quantitative using a 5-point Likert scale such as self-reported level of health.

Food consumption frequency variables (fruit, vegetables, meat, dairy, cereals, pulses, and soft drinks) were used to compute the condensed validated version of IASE (Healthy Eating Index for the Spanish population) [34]. This index evaluates how frequently individuals consume foods recommended for daily and weekly intake as well as those meant for occasional consumption. It also assesses dietary variety, crucial for maintaining a healthy diet. Scores range from 0 to 73, with a score of 10 indicating behaviors aligned with recommendations set by the Spanish Society of Community Nutrition (SENC) [35]. The IASE score categorizes nutritional habits into three groups: 58.4 < IASE < 73 classified as “Healthy”, 36.5 < IASE < 58.4 as “Needs changes”, and IASE < 36.5 as “Unhealthy.”

Variables related to nutritional and health habits not covered by IASE were categorized on a 4-point Likert scale (from 1 = no or low frequency to 4 = maximum frequency) using the same categorization followed in previous articles [36,37]. Body mass index (BMI) and minutes of exercise were used as numerical measures. This categorization provides a structured assessment of health-related behaviors, offering a detailed understanding of the participants’ habits in these domains.

Finally, the variables related to possible symptoms of eating disorders included concern about feeling fat or getting fat, inability to control food intake or experiencing a feeling of shame after eating, and concern about body shape. These frequency variables were categorized using a 6-point Likert scale (6 = always, 5 = very frequently, 4 = frequently, 3 = occasionally, 2 = rarely, and 1 = never). The survey also explicitly inquired whether the participants had been diagnosed with any eating disorders. Specifically, respondents were allowed to choose between the options of anorexia nervosa, bulimia nervosa, binge eating disorder, bigorexia, orthorexia, several at once, and other.

### 2.6. Data Analysis

Upon collection, questionnaire data were organized into a dedicated database and meticulously examined to rectify errors and inconsistencies, with a particular focus on data entry issues and outliers. Furthermore, certain variables underwent categorization or calculation based on others. Extreme BMI values (below 14 and above 40) were excluded to ensure data robustness.

Discrete variables were presented as absolute values and percentages (prevalence), while continuous variables were summarized using mean and standard deviation. Descriptive and inferential statistical analyses were subsequently performed.

The normality of the data was assessed using the Lilliefors test (Kolmogorov–Smirnov) at a 95% significance level, which indicated a departure from normal distribution. As a result, non-parametric statistical tests (Mann–Whitney, Kruskal–Wallis) were employed to analyze continuous variables, and the Chi-2 test was used for categorical variables. Additionally, a Dunn test with post hoc Bonferroni correction was utilized to provide further insights into differences identified among multiple groups or pairs [38]. All statistical analyses and plots were conducted using Python 3.9.0.

## 3. Results

### Socio-Demographic Characteristics of the Sample

The final sample included 9692 Spanish individuals living in Spain aged 18–30 years, whose detailed descriptions can be found in Table 1.

Among the respondents (N = 9692), 95.83% reported no diagnosed ED and 4.17% (N = 404) claimed to have one or more diagnosed ED. Specifically, 1.04% (N = 101) reported to have been diagnosed with AN, 0.73% (N = 71) BN, and the rest (2.40%) with other types of EDs. The results regarding possible eating disorder symptoms for the whole sample are detailed in Table 2.

The results related to possible symptoms of eating disorders in the sample including only individuals diagnosed with AN or BN are shown in Table 3.

As can be seen by comparing Table 2 with Table 3, the percentages of respondents reporting obesophobia, lack of control over food intake, and concern about body image were higher in individuals with diagnosed EDs compared with the healthy respondents.

The relationships between body mass index, healthy nutrition index, and frequency of consumption of different food and drink groups were analyzed separately in three different groups of respondents according to ED diagnosed ((1) No ED diagnosed = healthy; (2) anorexia nervosa; (3) bulimia nervosa). Statistically significant differences were found for many of the variables explored (see Table 4). Specifically, the BMI was different between the healthy population and people with diagnosed AN or BN, while no differences were found between people with one or the other EDs. The IASE scores were different only between the healthy population and those with BN. The consumption of fried foods was different between all groups, while that of ready meals was different between the healthy population and people with AN and between people with AN and those with BN. In contrast, the consumption of ultra-processed foods was only found to be different between the healthy population and those with AN. There were no statistically significant differences between groups for fish consumption (see Table 4).

Observing the relationships between the three groups studied and the frequency of beverage consumption, no differences were found for the consumption of water, sweetened soft drinks, or juice. On the contrary, the consumption of coffee or energy drinks differed significantly between healthy people and those with AN or BN (see Table 4).

No statistically significant differences were found between the three groups with respect to sedentary lifestyles. However, people with BN did more exercise than healthy people. The degree of health was reported as higher by healthy people than by people with AN or BN. With regard to sleep habits, waking up rested did not differ significantly between groups, but healthy people reported sleeping longer than people with AN and with better sleep quality. The Supplementary Materials section shows the detailed results of Dunn’s test for each habit variable for the population groups studied.

When analyzing the social habit variables, no differences were found between groups for alcohol consumption or nightlife. Nevertheless, people with BN smoked more and people with AN seemed to get drunk more frequently than healthy people.

**Figure 1:**
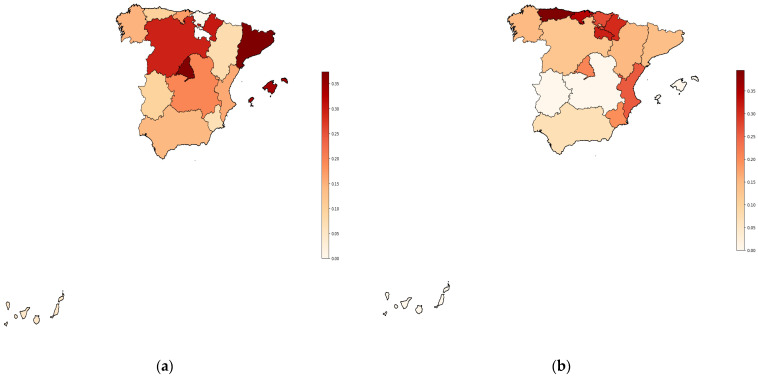
Prevalence of eating disorders according to regions in Spain. (**a**) Prevalence of anorexia per 100,000 people; (**b**) prevalence of bulimia per 100,000 people.

Regarding the socio-demographic variables (see Table 5), more females than males were represented in the sample (*p* < 0.001), and both AN and BN showed a significantly higher prevalence in women than in men. Specifically, all the cases with AN (N = 101, 1.04%) or BN (N = 71, 0.73%) in the sample were found among the women, while no men reported diagnosed EDs. No statistically significant differences between groups were found for the other socio-demographic variables explored: level of education, level of income, and size of municipality of residence. Finally, no significant differences (*p*-value = 0.45) were found in the prevalence of AN or BN between the different Spanish autonomous communities (Figure 1).

## 4. Discussion

The study aimed to describe the prevalence of eating disorder symptoms and their correlations with dietary and lifestyle habits in young Spanish adults aged 18–30 years old. The prevalence of EDs diagnosed in the respondents stood at 4.17% for all EDs, 1.04% for AN, and 0.73% for BN. In the sample studied, these percentages corresponded only to women, probably because in the general population, there is a higher prevalence of EDs in women [8], and most respondents were female. These numbers are in line with the recent review by Silén et al. [8] reporting that in Western countries, between 5.5 and 17.9% of young women and between 0.6 and 2.4% of young men had suffered from an ED in their adolescence. Specifically, 0.8–6.3% of females and 0.1–0.3% of males reported AN and 0.8–2.6% of females and 0.1–0.2% of males reported BN [8].

The comparison of the variables ‘concern about gaining weight’, ‘lack of control over food intake’, and ‘concern about body image’ between the subjects with diagnosed EDs and the entire sample may suggest that there are people who are prone to or close to developing a disorder or who even suffer from it but have not been diagnosed. For ‘healthy’ individuals (who have not reported as having been diagnosed with an ED), the percentage of ‘always’ or ‘almost always’ responses for the variable ‘fear of gaining weight ‘was around 25% and for the response ‘frequently’, it was more than 20%, adding up to about 46% of people who indicated that they worried, at least frequently, about gaining weight.

Frequent preoccupation with gaining weight or consistently experiencing a feeling of shame after eating are signs that may indicate the presence of an ED, particularly AN [39]. These behaviors may be associated with a distorted misperception of body image or a dysfunctional perfectionism. Such personality traits can lead to a persistent search for the ideal of thinness, constant measuring, and self-demanding, and this can be a triggering or maintaining factor in eating disorders, as they promote dieting, binge eating, and purging [40,41]. These can be hazardous, especially when combined with low self-esteem [42].

The variable “habitual lack of control over the amount of food consumed” could be correlated with eating disorders such as bulimia nervosa or binge eating disorders [42]. The latter occur when individuals consume large quantities of food in a short period, often in response to emotional stress, without feeling able to control their eating behavior. These binge eating episodes can lead to weight gain over time if not properly managed. On the other hand, if, after consuming a large amount of food, methods are used to eliminate what has been eaten, such as purging, vomiting, or laxatives, one may be dealing with BN [43].

For the variable ‘concern about body image’, there were around 30% of responses of ‘always’ or ‘almost always’ and close to 25% for the response ‘frequently’, adding up to more than 53% in the total sample. Body dissatisfaction is often cited as a critical risk factor for eating disorders. Individuals who are unhappy with their bodies are more prone to engage in disordered eating behaviors like bingeing and purging, aiming to attain satisfaction and conform to societal ideals of thinness [44].

These results, although by no means conclusive, suggest the need for larger population-based studies with the specific aim of detecting possible eating disorders in the Spanish young adult population, which may be more frequent than expected. Early intervention may play a crucial role in reducing the risk of pathology and long-term disability [45]. Particularly interesting is the high body image concerns found among respondents that could be a symptom or denote an onset of an ED, given that EDs arise due to a complex interplay of psychological factors, social pressures, and cultural expectations often related to body image and self-esteem [46].

Social media, advertising, and image culture have significantly impacted young people’s self-esteem, fostering a worrying decline in their emotional well-being. Constant exposure to unattainable beauty standards and success through digital platforms can generate constant and damaging comparisons, leading young people to feel inadequate and unwanted [47,48]. The pressure to conform to superficial ideals promoted by overblown advertising and image culture contributes to developing psychological complexes and adopting unhealthy habits [49]. Such constant shelling of idealized messages and representations could trigger eating disorders as young people desperately seek to achieve an unrealistic standard of beauty that ultimately affects their physical and mental health [50,51].

A higher prevalence of diagnosed EDs was found in women than in men, in line with numerous studies [6,52,53]. The interaction of various biological, psychological, and sociocultural factors can explain this gender disparity in the prevalence of eating disorders. Cultural and societal pressure on women about beauty standards, which prioritize extreme thinness, may lead to a greater likelihood of women developing eating disorders, as they feel obliged to conform to such unattainable ideals [54]. Gender role expectations also play a significant role. The ingrained perception that thinness is intrinsically linked to success and attractiveness may influence women’s attitudes and eating behaviors [55]. Biological and hormonal factors specific to women also come into play. It has been suggested that hormonal fluctuations associated with events such as puberty and the menstrual cycle may influence susceptibility to eating disorders. Although the exact relationship between these hormonal changes and eating disorders is still under investigation, their potential impact on women’s mental health is recognized [56,57]. Genetic vulnerability adds another layer to this complex equation. Although genetics is not the only explanation for the gender disparity in the prevalence of eating disorders, there is evidence that genetic predisposition may contribute to the higher incidence observed in certain demographic groups of women [58].

Living in a large city compared to a small town does not seem to affect the prevalence of EDs. Contrasting data can be found in the literature. A study by van Son et al. [59] found that urban life was a potential environmental risk factor for BN but not for AN, while a review conducted by Hay et al. [60] concluded that living in a metropolis was unlikely to be a significant risk factor in itself for developing an eating disorder. The same discordance of data on the benefits or risks of living in a city versus a rural area has been found for other diseases and disorders linked to diet and habits. For example, one study found that people living in major cities in Spain faced an increased risk of both overall mortality and mortality due to cancer compared to those residing in less urbanized areas [61], while another study showed evident territorial inequalities in mortality from diabetes mellitus in Spain [62].

Moreover, no significant differences were found in the prevalence of EDs between the autonomous community of residence. This may be attributed to several factors, as eating disorders are complex conditions that may be influenced by a combination of biological, psychological, social, and cultural factors that may be similar across the country. In fact, the autonomous communities in Spain share many cultural and social aspects including beauty standards and body ideals that may influence the prevalence of eating disorders. Although there may be regional differences in terms of eating habits, access to health care services, and stress levels, these variations may not be large enough to cause significant differences in the prevalence of EDs between autonomous communities [63,64,65].

Regarding the level of education and income of the respondents, no statistically significant differences were found in the prevalence of AN or BN by either of these determinants. Several factors could explain this homogeneity in prevalence. Firstly, the widespread availability of current health and nutrition information, facilitated by the Internet, social media, and newspapers, can help all individuals, regardless of their educational or economic background, to understand the risks associated with eating disorders and to adopt healthy practices [66,67]. Secondly, increased public awareness of eating disorders has led to greater early detection and help-seeking, which may be equally shared across all socio-economic groups [68,69]. Finally, the shared food culture in Spain, which values the Mediterranean diet and the importance of food as a social element, may act as a protective factor against eating disorders in all socio-economic strata [70].

The health factors and eating and lifestyle habits for the three population groups (healthy people, people diagnosed with AN, and those diagnosed with BN) showed that the mean BMI varied significantly (with a decrease of almost three points) between healthy people and people with AN, and a similar difference was found between people with BN and people with AN. This result is perfectly in line with what is known about AN and the extreme food restriction to which people with AN are subjected, with very low caloric intake leading to significant weight loss compared to healthy people [71]. In the sample with AN analyzed, this weight loss was not extreme, in fact, the mean BMI was within normal limits and only 24 (24%) people with this ED had a BMI below the value of 18, considered the limit for normal weight.

In contrast to AN, weight loss is not as evident in BN. Although individuals with BN may experience fluctuations in their weight, they usually maintain a weight within the normal range or may even be slightly above it. This is because, unlike the extreme food restriction seen in AN, people with BN tend to alternate episodes of excessive food intake (binge eating) with compensatory behaviors such as self-induced vomiting, laxative use, or excessive exercise. As a result, weight loss is not as pronounced in BN as in AN. However, weight fluctuations and compensatory behaviors can have severe effects on the physical and mental health of people with BN [72].

Regarding the dietary dimension, there were no significant differences between groups for the healthy eating index variable. The scores achieved for all three groups were within the range where changes are needed. With regard to specific food consumption, there were no differences between groups in the frequency of fish consumption, while there was a significant difference in the consumption of fried foods, fast food, and ultra-processed foods between healthy people and people with AN, with consumption by the latter being significantly lower. The reasons for these differences may be explained by the fact that people with AN have severe calorie control and these foods are often high in calories, fats, and sugars, which can lead to weight gain. People with AN often have extreme concern for their weight and shape, so they avoid eating foods that they perceive as “dangerous” for their weight control. On the other hand, people with AN often have excessive concern for their health and physical well-being. They may associate this food family with a poor diet and an unhealthy lifestyle, so prefer to avoid them. Finally, people with this condition may experience intense feelings of guilt or shame after consuming foods that are considered unhealthy or “forbidden”. Avoiding these foods is a protective mechanism to avoid such negative feelings [71].

Similarly, for the frequency of beverage consumption, there were no significant differences between groups in the consumption of water, sugary soft drinks, or natural fruit juices. Nevertheless, there was a higher consumption of coffee and energy drinks among people with AN and people with BN compared to the healthy population. Several reasons can be hypothesized to explain this difference in consumption patterns. These drinks may be used as appetite suppressants, and people with eating disorders may use them as a way to reduce their calorie intake and control their weight [73]. Caffeine can also have diuretic effects and stimulate metabolism, which some people may perceive as beneficial in controlling their weight or compensating for calorie intake [74]. On the other hand, caffeine in coffee and energy drinks can provide a temporary energy boost [75], which may appeal to people with AN or BN who suffer from fatigue due to food restriction, binge eating, and compensatory behaviors.

With respect to habits related to physical activity, no significant differences were found between any group for the sedentary lifestyle variable. However, bulimics spent more time per week doing physical activity than healthy people. However, it should be noted that for both groups, the average was above the 150 min per week recommended by the World Health Organization (WHO) as a healthy practice [76]. This difference could be derived from the fact that BN involves episodes of binge eating followed by compensatory behaviors, and excessive exercise could be one of the ways used to control weight or alleviate the guilt associated with binge eating [77]. No significant differences in sporting activity were observed for people with AN compared to the other two groups. This seems to be in contrast with most studies associating excessive physical activity with AN, as revealed in the systematic review by Rizk et al. [78]. These researchers highlighted how the voluntary increase in physical activity represented in some anorexic patients was a conscious strategy to maximize weight loss, while for others, the increase was involuntary and a consequence of weight loss, indicating that exercise may be under the control of a subconscious biological drive and involuntary cognition [78]. In this study sample, the physical activity of people with AN was not significantly higher than that of healthy people, perhaps because most of the population analyzed was already active and frequently practiced sport, so the difference was not high, and perhaps other strategies used by anorexics for weight control, such as extreme calorie restriction, stood out more.

As expected, significant differences were found between healthy people and anorexics or bulimics with respect to self-perceived health, indicating an awareness of people suffering from these EDs that their state of health is deficient. These patients are probably affected by some of the symptoms of these diseases such as, in the physical sphere, hair loss and muscle weakness or amenorrhea in women, and in the psychological sphere, emotional changes such as irritability, depression, or anxiety, although the questionnaire did not explore these dimensions [79].

With regard to quantity and quality of rest, people with AN slept significantly less than healthy people, and their quality of rest was also lower, as was the case for people with BN. These results confirm studies that have shown that insomnia is one of the consequences of these diseases. People with AN and BN may experience difficulty falling asleep or staying asleep during the night due to anxiety, preoccupation with food and weight or hormonal changes associated with these disorders, and episodes of hunger or purging during the night can disrupt sleep and lead to frequent awakenings. These changes in sleep patterns can lead to fatigue and daytime sleepiness, making it difficult for these patients to function normally [80,81].

### Strengths and Limitations

This study had some notable strengths. The main one lies in the large sample size that gives a reasonably accurate picture of the health behaviors of the young Spanish population. Another strength is the geographical representativeness of the sample, with data collected from all regions of Spain including the islands and from both large cities and rural settings. The breadth of variables explored during the study is another strength because it allowed us to relate the behaviors of different nutrition, sport, and lifestyle habits with socio-demographic determinants that can shed light on the health status of the young Spanish population.

The limitations included the data being collected through an online survey, which, although it allowed for an easy selection of the target population, may have presented a response bias. An attempt was made to minimize possible false or inaccurate answers by using a closed and anonymous questionnaire. Another limitation lies in the fact that respondents with eating disorders, both AN and BN, were all female. Although a higher prevalence of these illnesses in women is typical, the fact that there was no male representation in our sample prevents us from being able to make conclusions for this population group. Further studies are warranted focusing on EDs in the male population to complement the present study.

## 5. Conclusions

The issues related to EDs, particularly AN and BN, affect various segments of the population and are not exclusively confined to adolescents. The constant increase in the prevalence of these pathologies globally, partly attributable to the sociocultural changes experienced by Western countries in recent decades, has also been confirmed in Spain. The data emerging from this study indicate that the prevalence of EDs could be even higher than previously documented, considering that there are individuals whose behaviors reflects the presence of an ED, even in the absence of a formal diagnosis. It provides up-to-date and reliable data on these disorders and the factors influencing them in Spain, which may help to make public health decisions with a special focus on this age group. It would be of great interest to further explore how EDs may influence the dietary choices and lifestyle of the population, given the evident impact of such disorders on general well-being. Future studies should update and strengthen the research on eating disorders, considering the high risk of their evolution into social and health problems. Of paramount importance will be to better understand the prevalence and incidence of these disorders across various ethnic groups and social contexts to develop effective and tailored prevention and treatment strategies. A targeted and multidisciplinary approach is essential to effectively address eating disorders, thus contributing to the improvement of general well-being.

## Figures and Tables

**Table 1:** Socio-demographic characteristics of the sample (N = 9692) and individuals with AD and BD (N = 172).

	Mean; SD or N (%)	Mean; SD or N (%)
Male	1835 (18.93%)	0 (0%)
Female	7857 (81.07%)	172 (100%)
Age (years)	24.35; 3.36 (range: 18–30)	23.79; 3.19 (range: 18–30)
Male Age (years)	24.28; 3.40 (range: 18–30)	
Female Age (years)	24.37; 3.35 (range: 18–30)	23.79; 3.19 (range: 18–30)
	**Total**	
**Level of education**		
Basic education	3509 (36.21%)	72 (41.86%)
Higher education	6183 (63.79%)	100 (58.14%)
**Income level**		
Low	4878 (50.33%)	88 (51.16%)
Medium-high	3593 (37.07%)	65 (37.79%)
Don’t know-no answer	1221 (12.60%)	19 (11.05%)
**Municipality**		
<2000	479 (4.94%)	7 (4.07%)
2000–10,000	1643 (16.95%)	30 (17.44%)
>10,000	7570 (78.11%)	135 (78.49%)

Note: SD = Standard deviation.

**Table 2:** Symptoms of eating disorders (N = 9692).

	Never N (%)	Rarely N (%)	Occasionally N (%)	Frequently N (%)	Very often N (%)	Always N (%)
**Obesophobia**	1083 (11.17%)	1797 (18.54%)	2327 (24.01%)	2050 (21.15%)	1486 (15.34%)	949 (9.79%)
**No control**	1805 (18.62%)	2917 (30.10%)	2531 (26.11%)	1302 (13.43%)	869 (8.97%)	268 (2.77%)
**Body image**	291 (3.00%)	1483 (15.30%)	2764 (28.52%)	2389 (24.65%)	1673 (17.26%)	1092 (11.27%)

**Table 3:** Symptoms of eating disorders in individual with diagnosed anorexia or bulimia (N= 172).

	Never	Rarely	Occasionally	Frequently	Very often	Always
**Obesophobia**	1 (0.58%)	9 (5.23%)	23 (13.37%)	32 (18.60%)	43 (25.00%)	64 (37.61%)
**No control**	7 (4.07%)	26 (15.12%)	38 (22.09%)	32 (18.60%)	46 (26.74%)	23 (13.37%)
**Body image**	0 (0.00%)	2 (1.16%)	21 (12.21%)	35 (20.35%)	56 (32.56%)	58 (33.72%)

**Table 4:** Dietary and lifestyle habits in the healthy population and people with anorexia or bulimia.

Numerical Variable	Healthy	Anorexia	Bulimia	*p*-Value ^ζ^
**BMI**	22.93; 3.84	20.06; 2.48	23.13; 3.64	H-A (<0.001) H-B (1.00) A-B (<0.001)
**IASE**	53.42; 9.67	51.13; 10.52	50.67; 9.98	H-A (0.11) H-B (0.04) A-B (1.00)
**Fried food**	2.37; 0.84	1.90; 0.81	1.93; 0.78	H-A (<0.001) H-B (<0.001) A-B (<0.001)
**Fast food**	2.54; 0.74	2.13; 0.84	2.45; 0.81	H-A (<0.001) H-B (1.00) A-B (0.03)
**Ultra-processed food**	2.46; 0.93	2.12; 1.04	2.44; 0.95	H-A (<0.001) H-B (1.00) A-B (0.07)
**Fish**	1.77; 0.51	1.83; 0.63	1.73; 0.55	0.49 ^‡^
**Water**	3.44; 0.63	3.41; 0.75	3.49; 0.56	0.91 ^‡^
**Sugary soft drinks**	1.42; 0.66	1.37; 0.73	1.49; 0.79	0.23 ^‡^
**Juice**	1.27; 0.55	1.17; 0.47	1.13; 0.34	H-A (0.12) H-B (0.16) A-B (1.00)
**Coffee and energy drinks**	1.59; 0.66	1.77; 0.61	1.86; 0.76	H-A (<0.001) H-B (<0.001) A-B (1.00)
**Sedentary lifestyle**	1.66; 0.87	1.46; 0.76	1.55; 0.86	H-A (0.05) H-B (0.61) A-B (1.00)
**Self-perceived health**	3.86; 0.80	3.37; 1.05	3.41; 0.87	H-A (<0.001) H-B (<0.001) A-B (1.00)
**Sport**	172.58; 187.44	209.48; 219.29	221.09; 182.74	H-A (0.42) H-B (0.02)
**Sleeping hours**	2.67; 0.69	2.43; 0.78	2.45; 0.75	H-A (<0.001) H-B (0.03) A-B (1.00)
**Getting up rested**	2.55; 0.57	2.46; 0.66	2.49; 0.56	0.13 ^‡^
**Sleep quality**	3.52; 0.97	3.05; 1.00	3.24; 1.03	H-A (<0.001) H-B (0.046) A-B (0.59)
**Smoking**	1.21; 0.58	1.33; 0.72	1.44; 0.75	H-A (0.15) H-B (<0.001) A-B (0.36)
**Alcohol**	1.70; 0.77	1.57; 0.85	1.72; 0.90	0.07 ^‡^
**Getting drunk**	1.08; 0.32	1.03; 0.17	1.14; 0.35	H-A (<0.001) H-B (0.046) A-B (0.59)
**Night outings**	1.30; 0.51	1.24; 0.45	1.28; 0.57	0.36 ^‡^

Legend: ^ζ^ Kruskal–Wallis test with Bonferroni correction (pairwise comparisons); ^‡^ No significant differences between groups.

**Table 5:** Prevalence of eating disorders according to different socio-demographic groups.

	Healthy Population N (%)	Anorexia N (%)	Bulimia N (%)	*p*-Value ^ζ^
**Men**	1811 (19.50%)	0 (0%)	0 (0%)	<0.001
**Women**	7477 (80.50%)	101 (100%)	71 (100%)	
***p*** **-value ^Π^**	<0.001	<0.001	<0.001	
**High education**	5950 (64.06%)	60 (59.41%)	40 (56.34%)	0.53
**Low education**	3338 (35.94%)	41 (40.59%)	31 (43.66%)	
**Low Income**	4664 (57.50%)	45 (49.45%)	43 (69.35%)	0.02
**Medium–High Income**	3448 (42.50%)	46 (50.55%)	19 (30.65%)	
***p*** **-value ^Π^**	1.00	0.71	0.35	
**Small city**	462 (78.11%)	4 (83.17%)	3 (71.83%)	0.35
**Medium city**	1571 (16.91%)	13 (12.87%)	17 (23.94%)	
**Big city**	7255 (4.97%)	84 (3.96%)	51 (4.23%)	

Legend: percentages per column; ^ζ^ Chi-2 test; ^Π^ Adjusted *p*-value with Bonferroni correction.

## Data Availability

The data presented in this study are available from the corresponding author upon reasonable request.

## Supplementary Materials

The following supporting information can be downloaded at: https://www.mdpi.com/article/10.3390/medicina60101565/s1, STROBE checklist can be found in Supplementary Material. Figures S1–S10 show the results of Dunn’s test performed for each nutrition and lifestyle variable for the three population groups studied (healthy population, people with anorexia nervosa, and people with bulimia nervosa).
