# Supplementary material for: Influence of Nutrition, Lifestyle Habits, and Socio-Demographic Determinants on Eating Disorder Symptoms in the Spanish Young Adult Population: A Cross-Sectional Nationwide Survey
Source: Medicina (Kaunas). 2024 Sep 24;60(10):1565. doi: 10.3390/medicina60101565 (PMC11509460; doi:10.3390/medicina60101565)
Supplement: Supplementary file 1 [file medicina-60-01565-s001.zip › medicina-3203189-supplementary.pdf]

Supplementary Materials

Figures S1–S10 show the results of the Dunn's Test performed for each nutrition and lifestyle variable for the three population groups studied (healthy population, people with anorexia nervosa and people with bulimia nervosa).

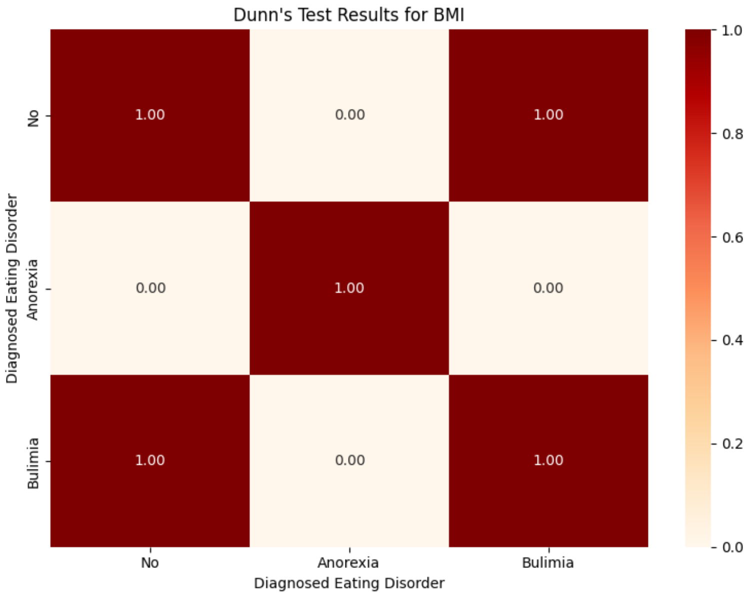

Figure S1: Dunn's Test Results for BMI.

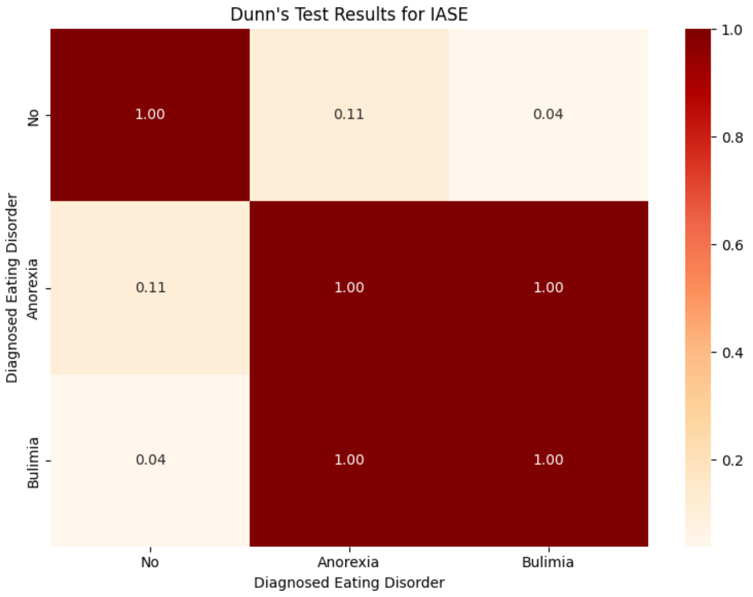

Figure S2: Dunn's Test Results for IASE.

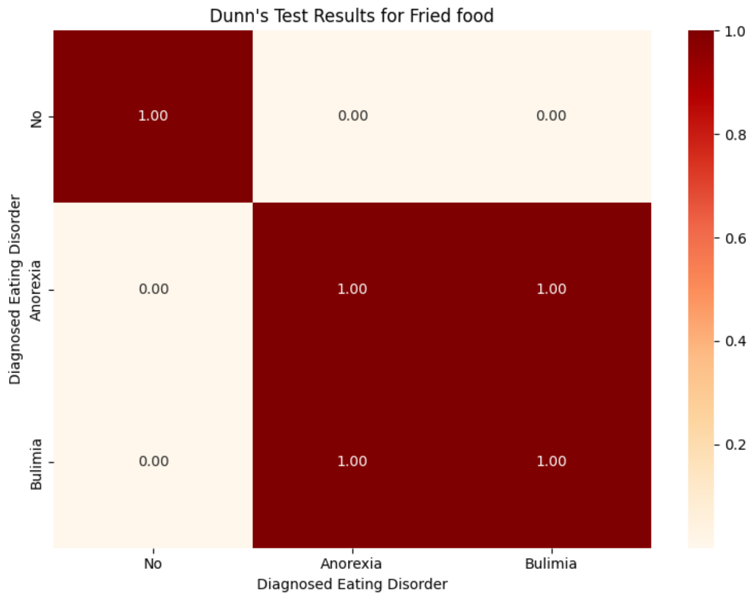

Figure S3: Dunn's Test Results for Fried Food.

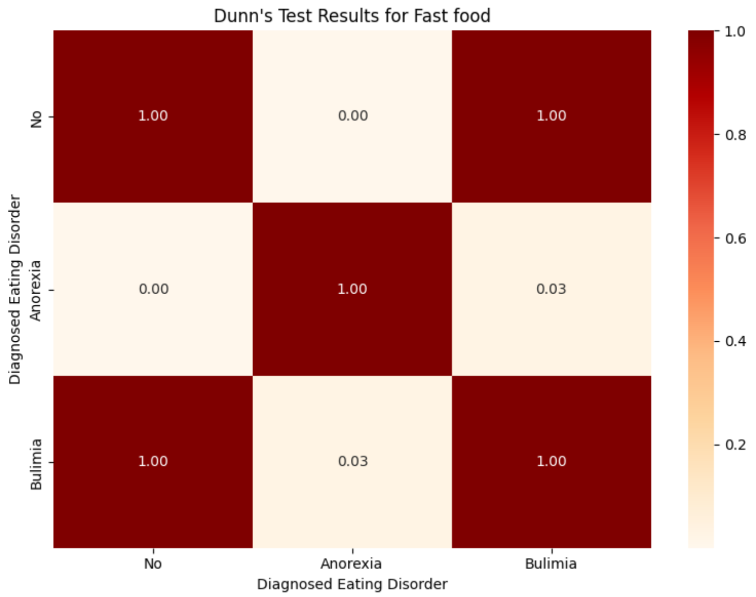

Figure S4: Dunn's Test Results for Fast Food.

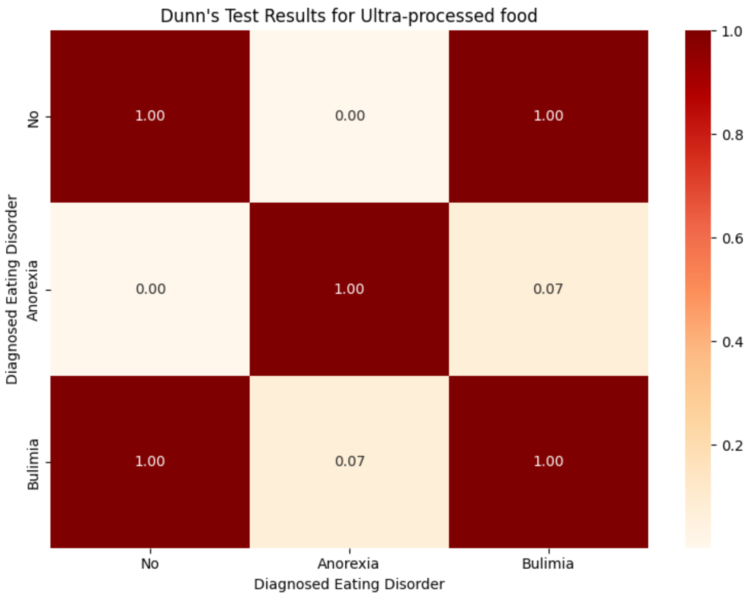

Figure S5: Dunn's Test Results for Ultra-processed Food.

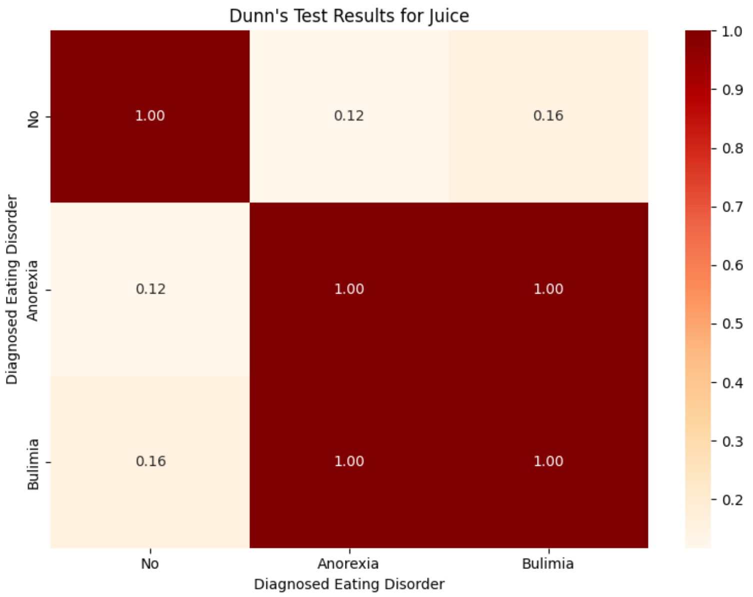

Figure S6: Dunn's Test Results for Juice.

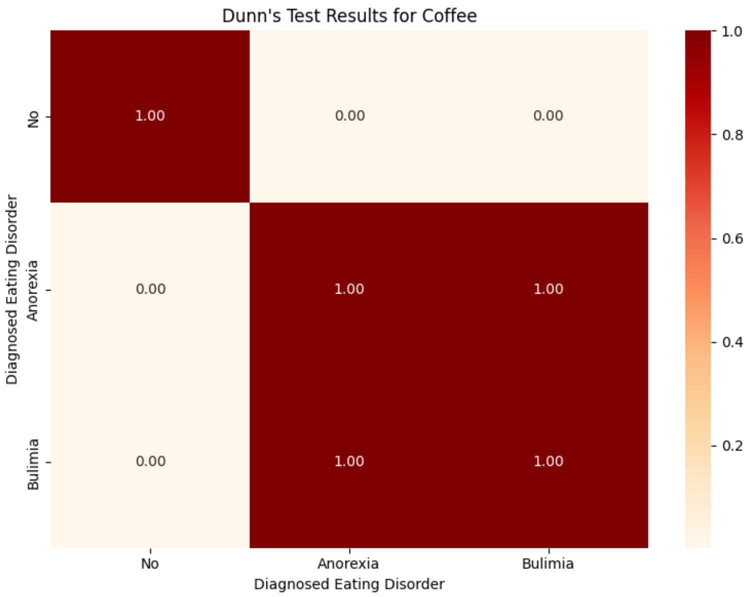

Figure S7: Dunn's Test Results for Coffee and Energy Drinks.

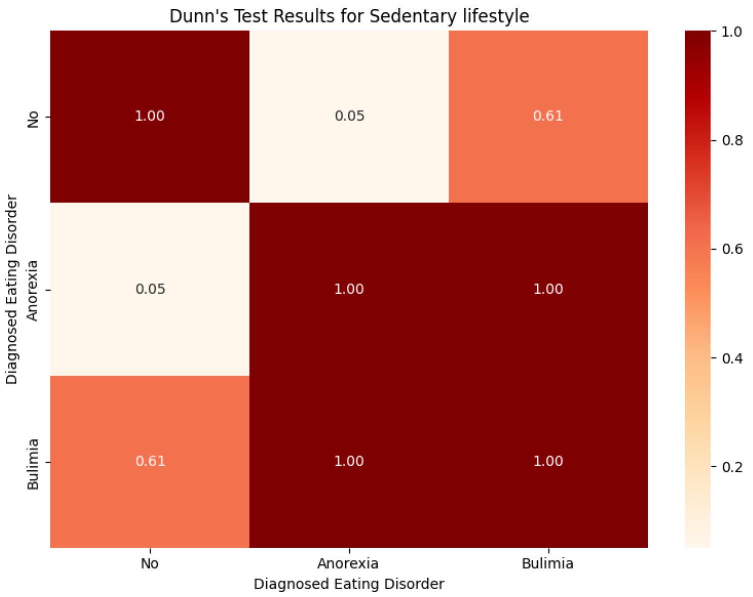

Figure S8: Dunn's Test Results for Sedentary lifestyle.

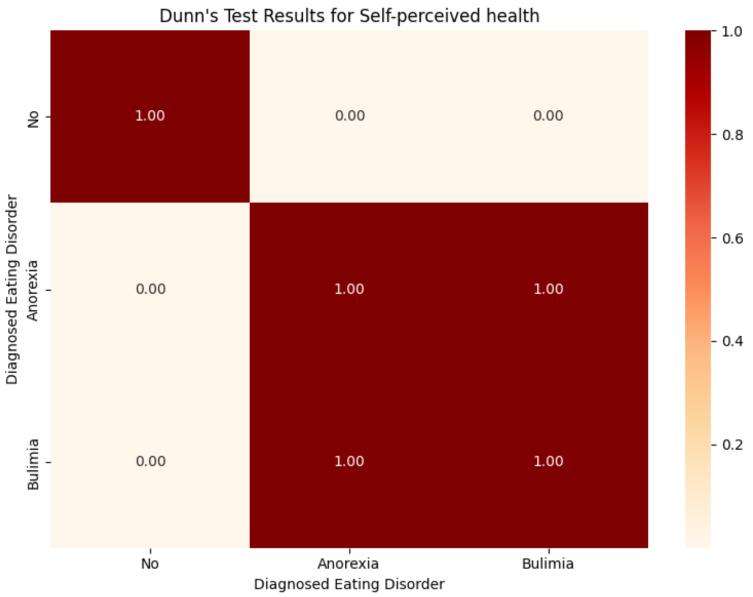

Figure S9: Dunn's Test Results for Self-perceived health.

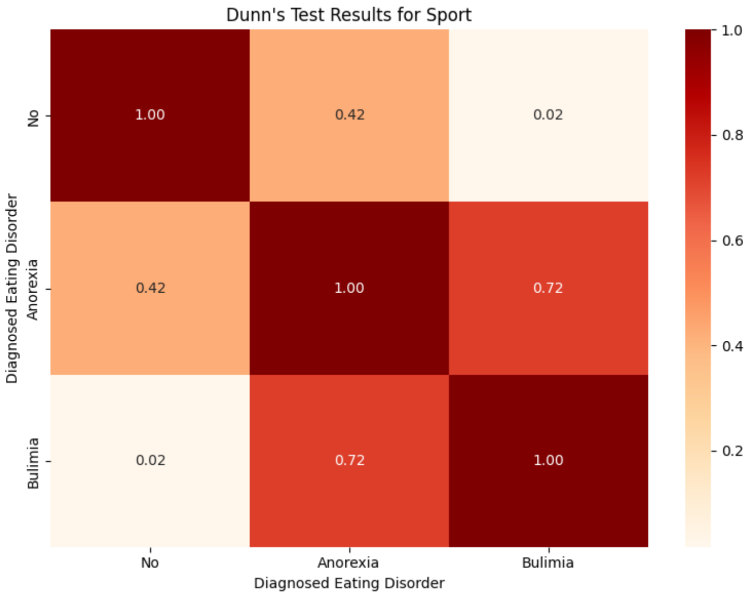

Figure S10: Dunn's Test Results for Sport.
